# Utility of stages of change construct in the planning of physical activity interventions among playgroup mothers

**DOI:** 10.1186/1756-0500-6-300

**Published:** 2013-07-29

**Authors:** Carlie Jones, Jonine Jancey, Peter Howat, Satvinder Dhaliwal, Sharyn Burns, Alexandra McManus, Andrew P Hills, Annie S Anderson

**Affiliations:** 1 Western Australian Centre for Health Promotion Research, School of Public Health, Curtin University, Western Australia, Perth, Australia; 2 Centre for Behavioural Research in Cancer Control, Curtin University, Western Australia, Perth, Australia; 3 Curtin Health Innovation Research Institute, Curtin University, Western Australia, Perth, Australia; 4 Mater Mother’s Hospital, Mater Medical Research Institute and Griffith Health Institute, Griffith University, Queensland, Nathan, Australia; 5 Centre for Public Health Nutrition Research, University of Dundee, Dundee, UK

**Keywords:** Physical activity, Mothers, Stages of change, Exercise

## Abstract

**Background:**

The objective of this research was to assess the physical activity levels among a unique cohort of Western Australian (WA) mothers with young children who attend a WA Playgroup. Associated factors were also investigated, including self-efficacy for physical activity, social support for exercise, relevant socio-demographic correlates, as well as the stages of change construct within the Transtheoretical Model (TTM).

**Results:**

421 women completed a questionnaire assessing physical activity behaviours. Of these, 368 participants completed the relevant physical activity evaluation items. 82.5% and 17.5% of the sample were classified as active and inactive, respectively. Associations between physical activity status and exercise stage of change were found. Additional associations were established for partner support and self-efficacy for physical activity.

**Conclusion:**

The majority of the sample was classified as active. Despite the high percentage of active participants, this study confirms the usefulness of the stages of change measure in that it can be utilised by health promotion practitioners to report physical activity behaviour and develop appropriate intervention strategies among a time poor and hard to reach population. Specifically the results are relevant to mothers in over 16,000 WA families who are involved with Playgroup WA programs. Interventions aimed at improving physical activity levels in mothers with young children should also consider the need to improve self-efficacy and social support.

## Background

Adverse health outcomes directly related to low levels of physical activity [1] are well recognised with the World Health Organization (WHO) listing physical inactivity as the fourth highest risk factor contributing to mortality on a global scale [2]. As a means of reducing the risk of chronic disease, the National Physical Activity Guidelines for Adults (1999) recommend Australians accumulate a minimum of 30 minutes of physical activity at a moderate-intensity level on most days of the week [3]. This guideline is also supported by WHO (2010) which recommends adults participate in aerobic activity at a moderate-intensity level for at least 150 minutes each week [2]. Alternatively, adults can complete at least 75 minutes of aerobic activity at a vigorous-intensity level, or a comparable amalgamation of both [2]. The 2008 physical activity guidelines for Americans mirror that of the WHO [4].

The need to achieve and maintain the recommended levels of physical activity is particularly relevant to women in the childbearing years [5]. Research indicates that this period is commonly associated with an increase in body weight and can lead to women experiencing long-term overweight and obesity [6,7]. Studies reporting on postpartum weight indicate that on average, women tend to lose weight up until 12-months post-partum [8,9], then increase their weight after this period [8]. This increase in weight following the initial weight loss period has been attributed to an increase in body fat [9,10], commonly caused by an energy imbalance where there is an increase in energy consumption and inadequate physical activity [9,11].

Physical activity behaviours of women during the reproductive years are less than optimal, with 75.3% of Australian females aged 18 to 44 years in 2007–8 classified as having low or sedentary levels of physical activity [12]. Among Australian women in this age category, only 19.1% and 5.6% were classified as having moderate and high levels of physical activity, respectively [12]. Miller et al. [13] noted that among Australian women aged 18 to 22 years, only 46% of those with children participated in sufficient levels of physical activity compared to 56% of those without children. Internationally in 2007, similar results were reported with only 51.6% of women aged 18 to 44 years in the United States of America achieving the recommended physical activity levels [14].

Many women undergo changes when they become mothers with their physical activity habits often shifting from being structured and intense to incidental and less intense [15]. Women with children also reported that it was more difficult to pursue physical activity for leisure and transport than women without children [16]. Nevertheless, women with children did express that physical activity associated with completing domestic duties was considered to be more manageable.

Mothers with young children, particularly those who were married and did not work outside the family home, were more likely to be classified as inactive [17]. Many barriers have been cited as preventing mothers from participating and maintaining adequate physical activity levels [15] including feelings of fatigue [15,18], and a perceived lack of time [15,18,19] and motivation [15,19]. Mothers also reported difficulty in managing the demands involved in raising young children such as organising their children’s sleep and feeding times around their personal physical activity [15,18,20]. Feelings of guilt associated with mothers taking time to exercise were also reported as a major barrier along with a change in priorities [15,21,22]. Commonly, mothers often prioritised their family’s needs before their own [15].

As mothers are primary role models for their offspring it is important that they maintain sufficient levels of physical activity not only for their continuing health and wellbeing but also for their children’s. Moore et al. [23] reported that children of active mothers were twice as likely to be active when compared to children with inactive mothers. Similarly, Hesketh et al. [24] reported that mothers who were more active, had greater intentions and self-efficacy for encouraging active play with their young children.

The aim of this paper was to assess the physical activity levels among a unique cohort of Western Australian (WA) mothers with young children who attended a WA Playgroup. The playgroup setting provides an avenue to reach over 16,000 WA families who are involved with Playgroup WA.

Specifically, this study analysed a component of a health behaviour change model and factors associated with physical activity levels. This involved an investigation of the stages of change construct within the Transtheoretical Model (TTM) [25-27] and reported levels of physical activity. Self-efficacy [28,29] and social support [13], along with relevant socio-demographic correlates, were also analysed.

## Methods

### Participants

Mothers with young children were recruited from WA playgroups registered with Playgroup WA (Inc) located within 20 kilometres of the Perth metropolitan area. Playgroup WA (Inc) supports community playgroups that offer opportunities for families with young children to play and learn in a fun and informal setting. Participants were required to have a basic proficiency in spoken English and to be the primary female caregiver of a child attending a registered playgroup. Individuals were excluded from the study if they had a medical condition that may have contraindicated their participation in the study, and/or were less than 18 years of age. In total, 421 women completed a base-line questionnaire that assessed dietary and physical activity habits. Of these, 368 participants completed the questions assessing physical activity levels. Ethics approval was granted by the Human Research Ethics Committee at Curtin University prior to the commencement of the study (HR171/2006).

### Procedure

Each playgroup was managed by a Playgroup Leader, a mother who volunteers to be responsible for the management of the playgroup. The Playgroup Leaders were contacted and invited to participate. As each playgroup varied in the number of playgroup sessions, the invited participants attended the session managed by the leader. An information sheet, consent form and self-report questionnaire were then provided to each consenting participant. Pre-paid return envelopes were provided to allow each participant to return their completed questionnaire.

### Measures

Questions regarding physical activity were derived from the International Physical Activity Questionnaire-Short Version (IPAQ-SV) [30] and items used by the WA Department of Health [31]. The self-report instrument enabled physical activity via walking to be assessed and separated into the domains of walking for errands and transport, and for recreation and exercise. All other forms of physical activity were included as moderate and vigorous-intensity physical activity. Moderate-intensity physical activity was defined as ‘…activities that take moderate physical effort and make you breathe somewhat harder such as swimming, jogging, cycling, lifting light loads or housework’ [30]. Vigorous-intensity physical activity was defined as ‘…activities that take hard physical effort and make you breathe much harder than normal such as heavy lifting, digging, aerobics and fast cycling or running’ [30]. Only activities that were completed for at least 10 minutes continuously were included. Physical activity levels for each participant were then calculated based on the methods recommended by Brown and Bauman [32] and replicated by Bell and Lee [17]. This involved an indirect assessment of energy expenditure where the metabolic equivalent of task (MET) was multiplied by the length of the physical activity in minutes [32]. The calculation used for each participant included multiplying the reported minutes of walking by three MET, moderate activity by four metabolic units, and vigorous activity by 7.5 MET and then totalling the final three figures thus creating a MET.mins score [17]. Based on the calculated score, participants were categorised as either inactive with a score of 599 or lower, and active if they had a score of 600 or more [17]. A score of 600 or more is equivalent to at least 30 minutes of daily moderate-intensity activity on five days over the course of a week [32], a level of activity reported to be adequate for health benefits [2,3]. The use of this method is reported to reduce the risk of underestimating the physical activity levels among women [32]. Using the same calculation method, a second definition for physical activity status was determined and used to categorise participants above or below the median physical activity score of 1395.

The stage of change construct within the TTM is commonly used to identify an individual’s stage in regard to their physical activity participation [25-27]. The exercise stage of change construct provides a series of stages that people can move through sequentially as well as intermittently. The five stages of change within the construct typically include pre-contemplation (Stage one, I currently do not exercise and I do not intend to start exercising in the next six months), contemplation (Stage two, I currently do not exercise but I am thinking about starting to exercise in the next six months), preparation (Stage three, I currently exercise but not regularly), action (Stage four, I currently exercise regularly but I have only begun to do so within the past six months) and maintenance (Stage five, I currently exercise regularly and I have done so for longer than six months) [33,34]. These stages were included in the current study along with the addition of a relapse stage (Stage six, I have exercised in the past but I am not doing so currently) [35].

Physical activity self-efficacy was assessed using a modified version of the McAuley Self-efficacy for exercise scale [36] as well as additional items used by Miller et al. [13] that are specific to mothers with young children. In total 17 items were used to assess exercise self-efficacy. A score was calculated by summing the score from each of the 17 items and then dividing the total by 17.

Partner support and family/friend support for exercise were measured using items from Sallis (1987) and Miller et al. (2002) [13,37]. Two sets of five items were used to assess each type of support (partner support and friend/family support). An overall score for both partner support and family/friend support was calculated based upon the mean response over the five items. A higher score for example reflected greater support in that domain.

Socio-demographic items used by the Australian Bureau of Statistics were also included in the analysis. These included mother’s age, marital status, household income, employment status, education status, country of origin, socio-economic level (SES) [38] (as determined by socio-economic indexes (SEIFA) for area) and parity. In addition measures of body mass index (BMI), breastfeeding and pregnancy status were included.

### Statistical analysis

The representativeness of the study sample was assessed by comparing the demographics to the Perinatal Statistics in Western Australia 2008 report [39] using the chi-square goodness-of-fit test. The association between personal, behavioural and socio-demographic factors, and activity status were assessed using chi-square test, logistic regression and independent samples t-tests. Activity status was defined in two ways: active and not active; below and above median. P-values < 0.05 were considered to be statistically significant. The data was analysed using Statistical Package for Social Sciences (SPSS, Version 18).

## Results

### Sample demographics

The mean age of the participants was 34.6 years (range 21–47 years). Among the sample, 73% (n=299) had more than one child; 97% (n=406) were married; 69% (n=291) were born in Australia and 58% (n=246) were not working outside the home at the time of the study. The demographics of the study sample were compared to the WA Perinatal Statistics in Western Australia 2008 demographic data [39] (Table 1). The study sample was representative for country of origin and area of residence. Statistically significant differences between the study sample and the Perinatal Statistics in Western Australia were observed for the variables mothers’ age, marital status, parity and socio-economic differences. The current study sample had a greater proportion of women who were older, married, had two or three children and came from a higher socio-economic level.

**Table 1:** **A comparison of the sample demographics with the perinatal statistics in Western Australia 2008 report**[39]

**Demographics**	**Study sample (N=421)**	**Perinatal statistics in Western Australia 2008 (N=30,234)**
**Mother’s age in years**		
18-24	6 (1.5%)	5882 (19.8%)
25-29	48 (11.7%)	8162 (27.4%)
30-34	134 (32.5%)	9211 (30.9%)
35-39	174 (42.2%)	5475 (18.4%)
40-44	45 (10.9%)	993 (3.3%)
45+	5 (1.2%)	39 (0.1%)
**Country of origin**		
Australia	291 (69.3%)	20852 (71.1%)
Other	129 (30.7%)	8471 (28.9%)
**Marital status**		
Married / Defacto	406 (97.1%)	27137 (89.8%)
Other	12 (2.9%)	3097 (10.2%)
**Parity**		
1 child	113 (27.4%)	12478 (41.3%)
2-3 children	286 (69.4%)	14830 (49.1%)
4-5 children	13 (3.2%)	2387 (7.9%)
6+	0 (0%)	539 (1.8%)
**Area of residence (WA)**		
North metropolitan	226 (53.6%)	12118 (52.5%)
South metropolitan	196 (46.4%)	10942 (47.5%)
**Socio-economic quintile**		
I	93 (22.6%)	7268 (24.5%)
II	125 (30.3%)	4382 (14.7%)
III	92 (22.3%)	5840 (19.7%)
IV	46 (11.2%)	6431 (21.6%)
V	56 (13.6%)	5792 (19.5%)
**Education**		
Completed year 10	19 (5%)	
Completed year 11	16 (4%)	
Completed year 12	47 (11%)	
TAFE/Diploma	112 (26%)	
University degree	212 (50%)	
Other	13 (3%)	
**Employment**		
Part-time	114 (27%)	
Full-time	6 (1%)	
Casual	45 (11%)	
Student	8 (2%)	
Currently not working outside the home	246 (58%)	

Note: Socio-economic quintiles are based on the index of relative socio-economic advantage and disadvantage produced by the Australian Bureau of Statistics, 2008 [38]. Group V depicts the lowest socio-economic status and group I represents the highest [39].

### Associations of personal, behavioural and socio-demographic factors with level of activity

The physical activity status (using the active or inactive definition) of study participants was compared with the following variables (Table 2): mother’s age, marital status, level of education, employment status, household income, parity, country of origin, socio-economic level, BMI, and breastfeeding and pregnancy status. Using the chi-square goodness-of-fit test, there were no statistically significant differences (P>0.05) between active and inactive mothers nor between mothers above and below the median physical activity score.

**Table 2:** A comparison of active and inactive participants

**Demographics**	**Inactive (N=64)**	**Active (N=304)**
**Mother’s age in years**		
18-29	9 (14.3%)	37 (12.4%)
30-39	44 (69.8%)	228 (76.5%)
40+	10 (15.9%)	33 (11.1%)
**Country of origin**		
Australia	46 (71.9%)	209 (69%)
Other	18 (28.1%)	94 (31%)
**Marital status**		
Married / Defacto	56 (88.9%)	264 (87.4%)
Other	7 (11.1%)	38 (12.6%)
**Parity**		
1 child	16 (26.2%)	82 (27.3%)
2+	45 (73.8%)	218 (72.7%)
**Socio-economic quintile**		
I	36 (57.1%)	166 (56.1%)
II	12 (19%)	51 (17.2%)
III	4 (6.3%)	23 (7.8%)
IV	8 (12.7%)	42 (14.2%)
V	3 (4.8%)	14 (4.7%)
**Education**		
Never attend, year 7–12 or other	16 (25%)	65 (21.5%)
TAFE/Diploma	11 (17.2%)	81 (26.8%)
University degree	37 (57.8%)	156 (51.7%)
**Income**		
<$18,200 to $51,999	7 (11.1%)	31 (10.6%)
$52,000 to $62,339	6 (9.5%)	12 (4.1%)
$62,400 to $72,799	6 (9.5%)	35 (11.9%)
$72,800 to $88,399	6 (9.5%)	34 (11.6%)
$88,400 to $103,999	13 (20.6%)	52 (17.7%)
$104,000 to $129,999	13 (20.6%)	51 (17.4%)
>$130,000	12 (19%)	78 (26.6%)
**BMI**		
Underweight (<18.5)	1 (1.6%)	10 (3.6%)
Healthy Weight (18.5-24.9)	34 (55.7%)	142 (50.9%)
Pre-obese (25–29.9)	9 (14.8%)	68 (24.4%)
Obese I (30–34.9)	6 (9.8%)	28 (10%)
Obese II (35–39.9) or Obese III (>40)	3 (4.9%)	6 (2.2%)
**Employment**		
Part-time or full-time	23 (35.9%)	83 (27.5%)
Casual, student or not working	41 (64.1%)	219 (72.5%)
**Pregnancy status**		
Pregnant	9 (14.1%)	25 (8.3%)
Not pregnant	55 (85.9%)	278 (91.7%)
**Breastfeeding status**		
Breastfeeding	9 (14.1%)	67 (22.1%)
Not breastfeeding	55 (85.9%)	236 (77.9%)
**Exercise stage of change**		
Stage 1 (Pre-contemplation)	8 (12.5%)	5 (1.7%)
Stage 2 (Contemplation)	21 (32.8%)	33 (10.9%)
Stage 3 (Preparation)	14 (21.9%)	54 (17.9%)
Stage 4 (Action)	6 (9.4%)	57 (18.9%)
Stage 5 (Maintenance)	5 (7.8%)	115 (38.1%)
Stage 6 (Relapse)	10 (15.6%)	38 (12.6%)

Note: Socio-economic quintiles are based on the index of relative socio-economic advantage and disadvantage produced by the Australian Bureau of Statistics, 2008 [38]. Group V depicts the lowest socio-economic status and group I represents the highest [39].

**Figure 1:**
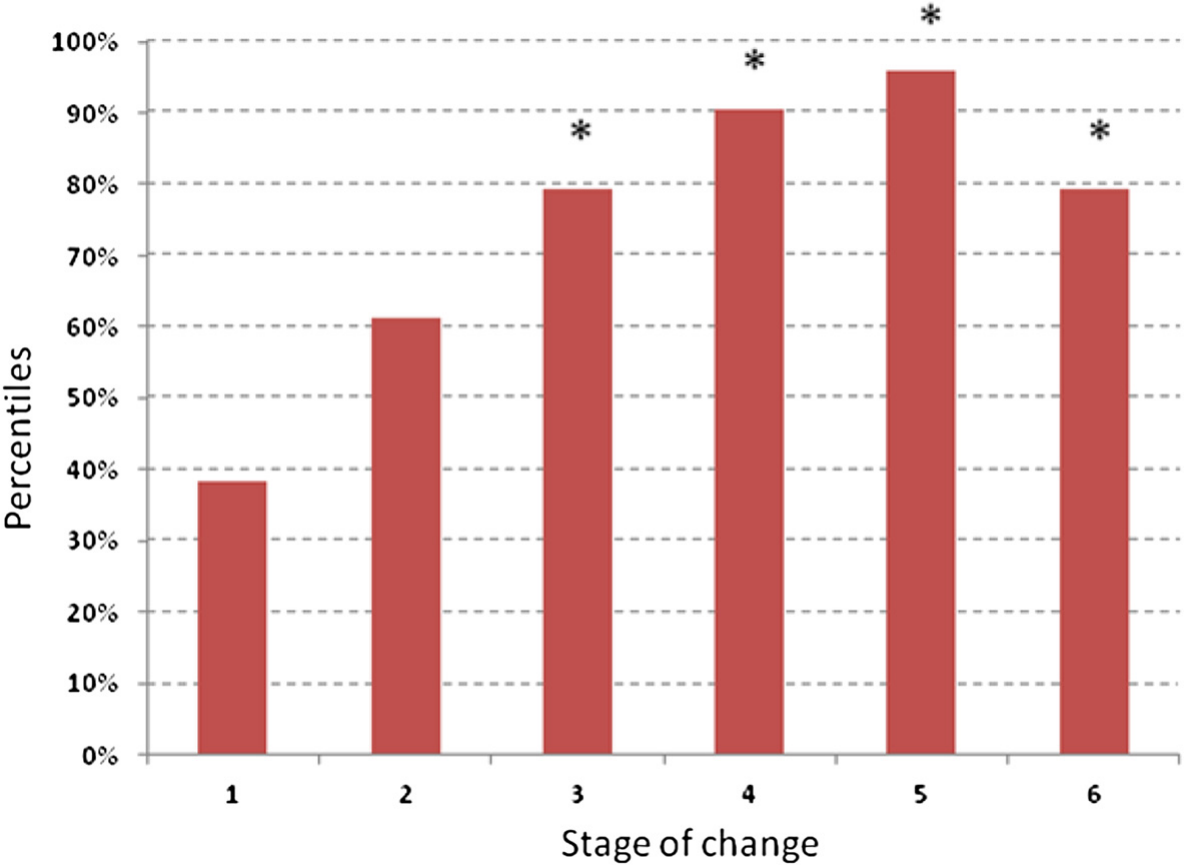
**Percentiles of physically active mothers with young children within Exercise Stages of Change construct.** Note: * denotes that the exercise stage of change was significantly different (p<0.01) compared to stage one.

When comparing physical activity status with the exercise stage of change variable, the logistic regression analysis indicated that stages three to six were significantly different from stage one (pre-contemplation) (P<0.01). Participants who were in stage three (preparation), stage four (action) and stage five (maintenance) were respectively 6.17 (95% CI: 1.76 – 21.81), 15.2 (95% CI: 3.75 – 61.55), and 36.8 (95% CI: 8.79 – 154.06) times more likely to be categorised as being active compared to those in stage one. Furthermore, participants who were in the relapse stage (stage six) were 6.1 (95% CI: 1.63 – 22.69) times more likely to be categorised as being active compared to those who were in the pre-contemplation stage. Associations with exercise stage of change and participants being above the median physical activity score (Audit score = 1395) were also found. Participants in stage four and five were 10.3 (95% CI: 2.08 – 50.42) and 10.2 (95% CI: 2.16 – 48.25) times more likely than participants in stage one to be above the median physical activity score. Figure 1 illustrates the percentiles of the sample within each stage of the construct.

Using independent samples t-tests, the results also demonstrated that participants’ self-efficacy and partner support scores was significantly associated with activity status when using both definitions for physical activity status (P<0.05) (active and not active; below and above median). Social support from friends and other family members, was statistically significant when using the ‘below and above the median’ definition (P<0.05).

## Discussion

This study covered a diverse sample of WA mothers with young children that included a greater proportion of women who were older, married, had two or more children and came from a higher socio-economic level when compared to a representative sample of WA mothers [39]. Physical activity status among participants was not statistically associated with level of education, income status, parity, SEIFA index, pregnancy and breastfeeding status or BMI when using both definitions for physical activity status. Interestingly, the current study showed no associations with marital status and employment, unlike Bell and Lee [17] who indicated that among a representative sample of 8545 Australian women, having children, being married and carrying out domestic duties full time was strongly linked to low levels of physical activity. They also reported that when analysing their data over time, moving in with a partner, getting married and having children was associated with reduced levels of physical activity. It is probable that the high percentage of women in the current study classified as being active is the reason for the lack of association with demographic variables; with 82.5% and 17.5% of the study sample classified as active and inactive, respectively. These figures represent higher physical activity levels than reported by the Australian Bureau of Statistics [12]. The high percentage of active women in the current study could be related to physical activity levels being overestimated when using the IPAQ-SV [40]. The majority of the sample in this study also self-reported a healthy BMI and a high SES status which could further explain the high levels of physical activity reported.

The age of women has previously been linked to the reported level of physical level [32]. Brown and Bauman [32] used MET.mins to calculate activity level and found that only 55.4% of women aged 30–44 years were adequately active, while 68.3% of women aged less than 30 years were adequately active. The cohort of mothers in the current study was mainly comprised of older mothers; nevertheless the data did not show any association between age and physical activity behaviour.

In the current study, associations between the exercise stage of change variable and physical activity level were identified. The exercise stage indicated by the participants was significantly associated with their physical activity level. For example, mothers who indicated that they were in the maintenance phase were 36.8 times more likely to be classified as being active than those in the pre-contemplation stage. In addition, mothers who were above the median physical activity score were more likely to attain a higher level in the Exercise Stages of Change Construct. In total, 48.2% of this sample reported being in the action or maintenance phase. A similar correlation was reported by Fahrenwald & Walker (2003) [41]. There is limited Australian data available to make comparisons, nevertheless the results from the current study indicate a more active sample than reported by Keller et al. [26] with only 13% of their cohort reporting regular participation in exercise. The current results also indicated that a high proportion of mothers were in the relapse stage, suggesting that while the majority of this sample were active, many struggled to maintain these levels due to competing demands when raising children.

Information about the current physical activity behaviour of participants and their stage of change is valuable. By utilising this construct during the needs analysis stage, the results can confidently assist program planners to develop and target relevant strategies by encouraging those in the maintenance stage to remain active and supporting those who are inactive to become active.

The results from this study also found links between self-efficacy for physical activity as well as partner support for physical activity. Mothers who had higher self-efficacy and partner support for physical activity were significantly more likely to be classified as being active compared to those who were inactive. These results were replicated when the second definition for physical activity status was included in the analysis (above or below the median physical activity score). Nevertheless, an association between social support from friends and other family members with physical activity was only found when using the ‘above and below the median’ definition. An association between social support for exercise and personal self-efficacy for physical activity has been discussed in previous research. Among a sample of mothers with young children, intervention strategies aimed at increasing partner support and self-efficacy were successful in helping to improve physical activity levels within the sample [13]. Hinton and Olson (2001) assessed psychosocial measures among women at 12-months post-partum and found that higher self-efficacy for exercise as well as a greater intention to exercise resulted in healthier levels of physical activity [42]. An association between self-efficacy and levels of physical activity was also reported by Fahrenwald et al. [28] and Miller and Brown [29]. By confirming an association between physical activity, social support and self-efficacy among a sample of mothers, practitioners working on interventions with these groups should aim to identify current support and self-efficacy levels and include intervention strategies that aim to improve these measures.

A limitation of the current study is that the MET.mins method can overestimate energy expenditure depending on age and weight [43]. The method in the current study was used to make comparisons to previous research [17,32]. Objective physical activity measurements for evaluation were not used in this study due to time and financial constraints as well as due to the barriers associated with the target group discussed in a previous publication [15]. Items used [30,31] have been recognised as being appropriate to assess physical activity levels [30].

The identified results are specific to mothers attending WA playgroups. With over 16,000 families’ involved with Playgroup WA, the playgroup venue provides an established and accessible setting where health professionals can easily access mothers and their young children.

## Conclusion

Despite having higher physical activity levels, it appears many mothers with young children do need to overcome various barriers to engage in regular physical activity. The results from this study confirm the usefulness of the stages of change measure in that can be utilised by health promotion practitioners to identify physical activity behaviour and inform appropriate intervention strategies, specifically in playgroup settings. Interventions aimed at improving physical activity levels in mothers with young children should consider the relevance of the Stages of Change Construct as well as the need to strengthen self-efficacy and social support.

## Abbreviations

WHO: World Health Organization; WA: Western Australian; TTM: Transtheoretical Model; IPAQ-SV: International Physical Activity Questionnaire-Short Version; MET: Metabolic equivalent of task; SES: Socio-economic level; SEIFA: Socio-economic indexes for areas; SPSS: Statistical Package for Social Sciences; BMI: Body mass index

## Competing interests

The authors declare that there are no competing interests.

## Authors’ contributions

CJ planned and implemented the research and wrote the first draft of the paper. JJ assisted with the planning of the research was the second reviewer of the paper. SD assisted with the planning and data analysis. PH, AM, ASA and APH assisted with the planning of the research and reviewed the paper. All authors read and approved the final manuscript.
